# Observation of dose-rate dependence in a Fricke dosimeter irradiated at low dose rates with monoenergetic X-rays

**DOI:** 10.1038/s41598-018-21813-z

**Published:** 2018-03-16

**Authors:** Mel O’Leary, Daria Boscolo, Nicole Breslin, Jeremy M. C. Brown, Igor P. Dolbnya, Chris Emerson, Catarina Figueira, Oliver J. L. Fox, David Robert Grimes, Vladimir Ivosev, Annette K. Kleppe, Aaron McCulloch, Ian Pape, Chris Polin, Nathan Wardlow, Fred J. Currell

**Affiliations:** 1 School of Maths & Physics, Queen’s University Belfast, University Road, Belfast, BT7 1NN UK; 2 Centre for Advanced and Interdisciplinary Radiation Research (CAIRR), Queen’s University of Belfast, Belfast, BT7 1NN Northern Ireland UK; 3 GSI Helmholtzzentrum für Schwerionenforschung GmbH, Darmstadt, 64291 Germany; 4 Department of Radiation Science and Technology, Delft University of Technology, Delft, 2629 JB The Netherlands; 5 Diamond Light Source Ltd., Harwell Science and Innovation Campus, Didcot, OX11 0DE UK; 6 Cancer Research UK/MRC Oxford Institute for Radiation Oncology, Gray Laboratory, University of Oxford, Old Road Campus Research Building, Off Roosevelt Drive, Oxford, OX37DQ UK; 7 Institute of Molecular Sciences (ISMO), UMR 8625, University Paris-Saclay, Université Paris Sud, CNRS, 91405 Orsay Cedex, France

## Abstract

Absolute measurements of the radiolytic yield of Fe3+ in a ferrous sulphate dosimeter formulation (6 mM Fe2+), with a 20 keV x-ray monoenergetic beam, are reported. Dose-rate suppression of the radiolytic yield was observed at dose rates lower than and different in nature to those previously reported with x-rays. We present evidence that this effect is most likely to be due to recombination of free radicals radiolytically produced from water. The method used to make these measurements is also new and it provides radiolytic yields which are directly traceable to the SI standards system. The data presented provides new and exacting tests of radiation chemistry codes.

## Introduction

The accurate determination of radiolytic yields underlies all radiation research. The applications of such research include novel cancer therapy options, innovative industrial chemical process and the management of nuclear waste products^1–5^.

Previous work on the determination of radiolytic yields (G-values) with synchrotron sources used soft X-rays deposited in thick samples^6^, resulting in a large dose rate variation along the sample’s depth. The work of Hoshi *et al*.^7^ investigated the G-values for a ferrous sulphate dosimeter using a similar technique. In contrast, the measurements reported here were made using thin samples compared to the attenuation length of the radiation and hence the variation in dose and doses rate throughout the irradiated region is much smaller, leading to better defined experimental conditions. In this work, the power absorbed was measured to determine the total energy absorbed by the sample. Also, automated irradiation allows for higher throughput of samples. The results obtained could be used to benchmark computer models of radiation chemistry^8–13^ that will be used in applications of radiation research. This method was benchmarked using a variation of the standard chemical dosimeter, the ferrous sulphate dosimeter, which is used in most basic radiation research to measure energy deposited with irradiation (dose)^5,14–18^ and has been used as a dosimeter on the beamlines used in this study^1^.

In this paper we used the radiation chemistry research platform of Polin *et al*.^19^, and an add-on developed to enable a new, accurate method for the measurement of radiolytic yields in thin liquid samples. Essentially, photons from a synchrotron x-ray source pass through a thin-walled sample chamber that holds a known volume of sample with the attenuated beam being detected by a calibrated photodiode downstream of the sample chamber. By comparing the photodiode signal with and without sample in place, the absolute power absorbed by the sample during irradiation is deduced. From this a dose rate is calculated taking into account the time structure of radiation production of the synchrotron x-ray source, which is a pulsed x-ray source^20^.

## Results and Discussion

### G-values as a function of dose rate

**Figure 1:**
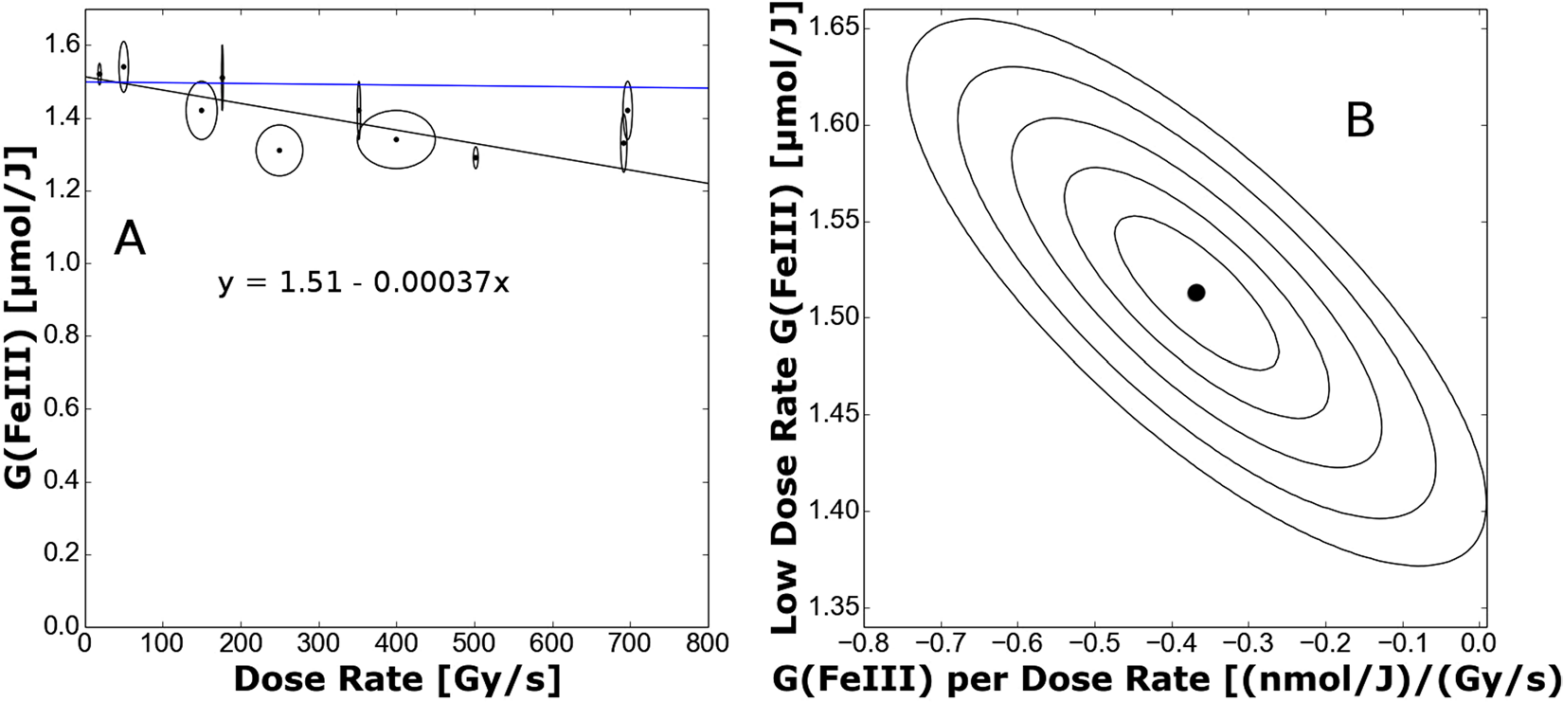
Total least squares fit of the data. Part A showing the best linear fit plotted (*c* = 1.51 ± 0.06 *μ*mol/J and *m* = −0.37 ± 0.09 (nmol/J)/(Gy/s), as indicated on the graph) against the data and error bars (plotted as ellipses). In part B, there is a contour plot of the likelihood that a fit fits the data. Each contour is about a standard deviation further away from the best fit, which is indicated by the point in the center of the contours^21^. The intercept of the best fit line agrees well with the model by Yamaguchi^22^. The blue line shows the predicted change in G-value due to spectral hardening effects. With an intercept at 1.498 *μ*mol/J and a slope of −2.11 × 10^−2^ (nmol/J)/(Gy/s), i.e. two orders of magnitude smaller than the observed effect.

**Figure 2:**
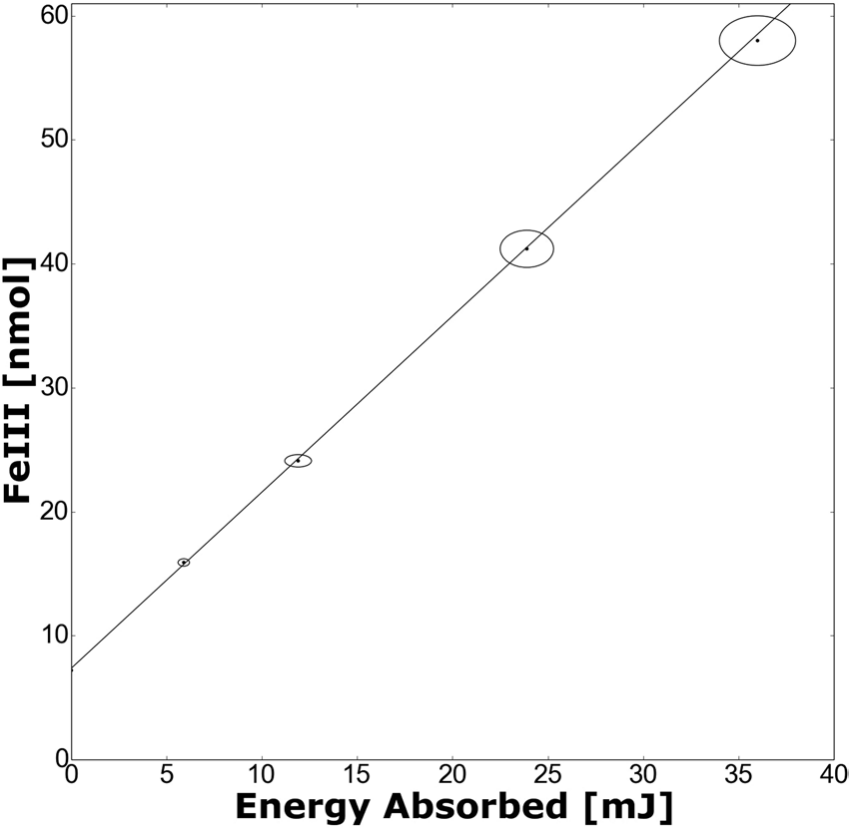
Measured amount of Fe^3+^ as a function of radiation energy absorbed by the sample. The slope of the line of best fit through the data is the G-value (1.42 ± 0.08 *μ*mol/J). Error bars (indicated by ellipses) are ± two standard errors and indicate statistical errors alone. These measurements were taken with a dose rate of 150 ± 20 Gy/s.

The variation of the G-value of Fe^3+^ in the ferrous sulphate dosimeter against the average dose rate during a pulse is seen in Fig. 1. Measurements were made at a photon energy of 20 keV (monoenergetic beam). Each data point is derived from a set of measurements like those shown in Fig. 2, made at a specific dose rate, the G-value being determined from the resultant slope. The line shown in Fig. 1 was determined using a total least squares linear fit (i.e. accounting for errors in both dose rate and G-value)^21^. At low dose rates the G(Fe^3+^) is 1.51 ± 0.06 *μ*mol/J. This value compares well to model values from Yamaguchi (1.4852 *μ*mol/J under these conditions)^22^ suggesting that the absolute G-values obtained are reliable. The G-value has been noted to be dose rate independent up to 20 kGy/s^5^. However, the results presented here show dose rate dependence in the G value of −0.35 ± 0.09 (nmol/J)/(Gy/s) for dose rates below 1 kGy/s. This previously unobserved dose rate dependence is only noticeable due to the sensitivity of the technique. It is noteworthy that the contours of likelihood of fit shown in Fig. 1 (right hand panel B) indicate the conclusion that there is a decrease in the G-value as the dose rate increases is valid with greater than 5-sigma confidence (i.e. it is an extremely robust conclusion).

### Consideration of artifactual effects

It is worth considering the source of this decrease in G-value as a possible experimental artifact. Initially, we might consider whether oxygen depletion plays a role in the observed effect. The presence of molecular oxygen in the medium is of particular importance^23,24^, mainly through the reaction

1 $${{\rm{e}}}_{{\rm{aq}}}^{-}+{{\rm{H}}}^{+}\to {{\rm{H}}}^{\bullet }$$

2 $${{\rm{H}}}^{\bullet }+{{\rm{O}}}_{{\rm{2}}}\to {{\rm{HO}}}_{{\rm{2}}}^{\bullet }$$

This reaction chain markedly increases the Fe^3+^ yield in the ferrous sulphate dosimeter– with no oxygen present in solution, the number of Fe^2+^ ions oxidized by H^•^ is reduced from three to one^24^, markedly reducing the yield. In an aerated solution, the yield after Co-60 *γ*-ray irradiation is typically 15.5 ± 0.2 ions per 100 eV^16,17,24,25^ versus only 8.2 ± 0.3 ions per 100 eV in an anoxic solution^16,17,24–27^. We expect oxygen to be steadily depleted over the course of irradiation, with each reaction down the oxic pathway removing a single molecule, and with higher dose rates increasing ionization and depletion rates. To investigate this effect we estimated the number density of oxygen molecules in the medium, through a method previously described^28^, and simulated the removal of oxygen through ionization events. However, this analysis suggested that oxygen depletion could not account for the observed effect, and was dependent on total dose received rather than the dose rate. The same analysis also predicted that the switch from oxic to anoxic reactions following oxygen depletion would occur rapidly rather than gradually, and indeed previous literature indicates a rapid switch in yield following oxygen depletion^5^, with no obvious dose rate effects up to 10 Gy/min^5,18^. Furthermore, if oxygen (or an other substance) is being depleted sufficiently to alter the dosimeter’s response then this would be reflected in a departure from the linear trend shown in Fig. 2. Statistical analysis of the dose dependences observed at different dose rates rules out consumption effects as is discussed in supplementary information.

**Figure 3:**
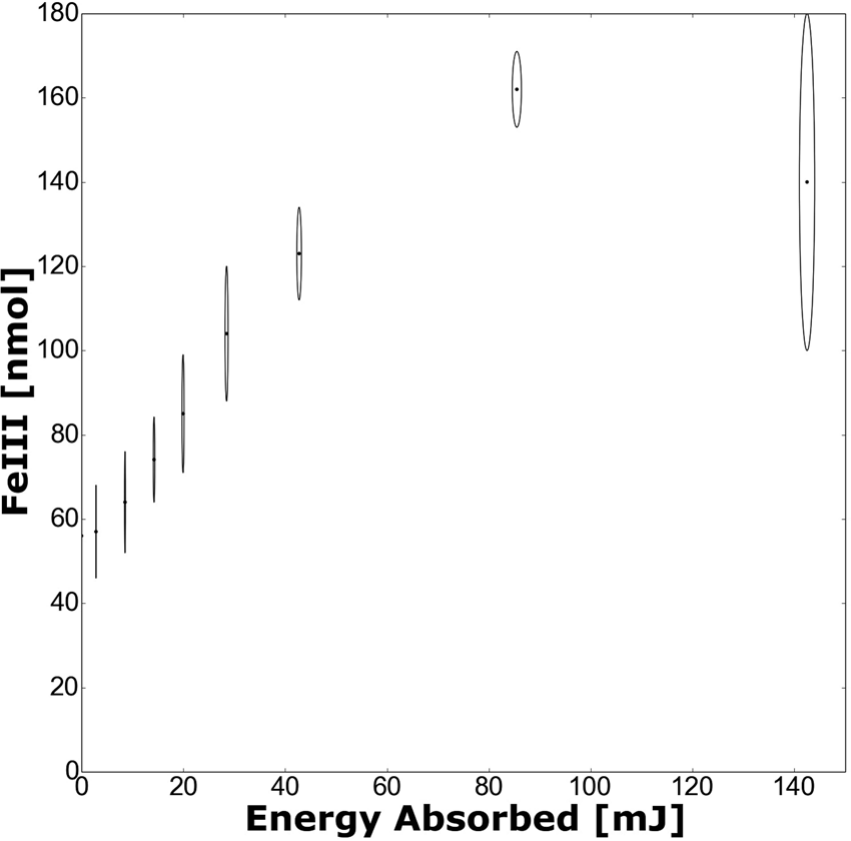
Measured amount of Fe^3+^ as a function of radiation energy absorbed by the sample; showing the turnover in production from the depletion of oxygen. Error bars are ± two standard errors and indicate statistical errors alone. These measurements were taken on B16^29,31^ with 16 keV X-rays (monoenergetic beam) at a dose rate of 575 ± 17 Gy/s.

To illustrate the depletion effect, we performed a similar experiment but at a lower photon energy (16 keV) thereby allowing us to achieve higher energy depositions in a reasonable time. As can be seen in Fig. 3, the lowering of the yield occurs at a higher deposited energy than the range over which the G-values were determined. Since the data shown in Fig. 2 and all other such plots (taken at different dose rates to derive the data shown in Fig. 1(A)) are linear across the whole dose range studied, depletion effects can not account for the effect observed.

Another possible mechanism responsible for this effect on the radiolytic yield is spectral hardening. This occurs because when aluminum attenuators are introduced they preferentially remove the lower photon energy x-rays. Higher photon energy x-rays produce a higher radiolytic yield^22^. As these higher energy photons form a greater and greater proportion of the spectrum the radiolytic yield will consequentially rise. The mono-energetic x-rays that irradiate the sample are selected from the x-ray spectrum produced, on I15 by a wiggler and on B16 by a bending magnet, followed by transmission through a monochromator. A RuB4C double multilayer mirror monochromator was used on B16^29–31^. A Si(111) double crystal monochromator (DCM) was used on I15. These monochromators can reflect, at a much lower intensity, higher photon energy photons that are an integer multiple photon energy of the selected photon energy (i.e. higher orders of light), as e.g. in case of DCM on I15^32^ the 3rd harmonic of fundamental/primary photon energy selected. The spectrum, S (*E*), produced by the double multilayer mirror monochromator on B16 was simulated (see supplementary information). The attenuated spectrum, *S*′(*E*), after attenuation by an aluminium with thickness (*t*) is calculated from the aluminium attenuation coefficients (*μ*_*Al*_) for each different photon energy (*E*), from equation 3^33^.

3 $$S^{\prime} (E)=S(E){e}^{-{\mu }_{Al}t}$$

The relative absorption as a function of the photon energy Δ(*E*), for this attenuated spectrum in a sample with thickness (x) using the sample’s energy-absorption coefficients (*μ*_*en*:*F*_), is given by equation 4^33^.

4 $${\rm{\Delta }}(E)=S^{\prime} (E)(1-{e}^{-{\mu }_{en:F}x})$$

The measured G-value, *G*_*m*_(*Fe*^3+^) by the radiation spectrum *S*′(*E*) can be determined by taking a weighted average of the energy dependent G-values G(Fe^3+^) taken from^22^, using equation 5.

5 $${G}_{m}(F{e}^{3+})=\frac{{\int }_{0}^{inf}{\rm{\Delta }}(E)G(E)dE}{{\int }_{0}^{inf}{\rm{\Delta }}(E)dE}$$

Using the spectrum shown in supplementary information, the variation in the measured G-values was determined using equation 5. The results of this simulation is depicted on Fig. 1 as the blue line. Since this line has a far lower (almost negligable) slope than the one observed, it is safe to conclude that spectral hardening is not the reason for the effect observed.

### Consideration of these effects as a result of recombination

Having considered the readily apparent sources of artifacts and shown that they are unable to account for the trend observed, we must consider alternate mechanics to explain this effect. While ferrous sulphate dosimeters have long been employed, the underlying radiation chemistry is in fact rather complex, with Monte Carlo simulations requiring in excess of 60 possible interactions to capture the observed dynamics of radiation with oxygenated water^25^, including radical interactions. Radical recombination may explain the observed effect. It is well known^4,15,34^ that recombination in heavy ion tracks acts to reduce the G-value of Fe^3+^ in the ferrous sulphate dosimeter. A similar effect has been observed at similar dose rates with electrons^35,36^. Recombination occurs when radicals interact with each other to produce non-radicals. Most of these non-radicals will be inert to oxidation pathways of Fe^2+^, reducing the G-value of Fe^3+^.

For any ionizing radiation, recombination is dependent on dose rate. As dose rates increase, radicals from separate primaries are produced closer together in time so recombination occurs more often. This is because radicals form and react within a reactive micro-zone around an ionization event called a spur^5,17^. At higher dose rates these spurs interact with each-other increasing the recombination rate.

The calculations of Hill and Smith^37^ are instructive since they consider the physical and chemical development of a complete track compared to the situation where the effect of short tracks (i.e. where isolated spurs are considered). In Fig. 1 of this paper, there is a clear difference between the G(Fe^3+^) as calculated with short and full tracks at 20 keV, with full tracks producing a lower G-value. Hence it is clear that spur-spur interactions act to significantly lower the G-value at this energy. As the dose rate increases, so too will the interactions between spurs originating from different primary photons. Accordingly the G-value is expected to lower, in line with our observations. These dose-rate effects are small at the dose-rates considered, which is why they have not been previously observed with x-rays, whereas the technique outlined has sufficient sensitivity to detect these dose-rate effects. This suggests the method outlined could improve understanding of water radiation chemistry, with the data set presented here (Fig. 1) providing new reference data against which computer codes simulating radiation chemistry^8–13^ can be validated. The synchrotron provides high dose rates with mono-energetic photon beams, hence giving stringent tests of such codes. However, this method is not limited to use on synchrotrons it can be used with any type of radiation source; provided means of establishing the power in the beam are available.

## Methods

### Sample handling

**Figure 4:**
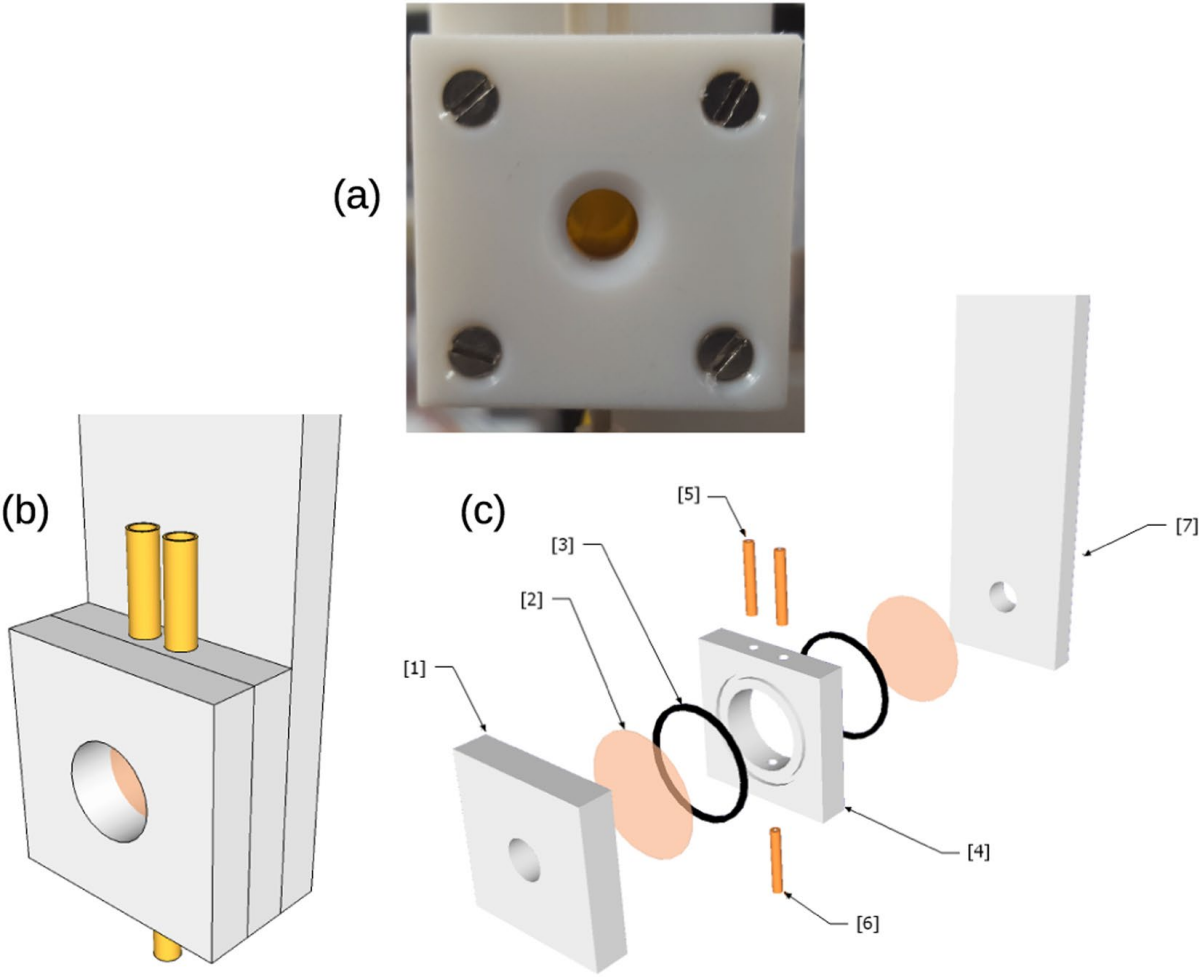
(**a**) Photograph of the chamber (**b**) Sketch of the chamber (**c**) An exploded view of the chamber showing its component parts^1^: front block^2^, kapton windows^3^, o-rings^4^, the chamber block^5^, Inlet PEEK tubing^6^, Outlet PEEK tubing^7^, Back block that is connected to supporting structure.

For the radiation chemistry research platform of Polin *et al*.^19^, an add-on was developed to enable the measurement of radiolytic yields in geometrically thin liquid samples. This add-on needs only two syringe pumps and a means to translate the sample catcher, so it could easily be implemented as a standalone system. Essentially, photons from a synchrotron X-ray source pass through a thin-walled sample chamber that holds a known volume of sample with the attenuated beam being detected by a calibrated photodiode downstream of the sample chamber. By comparing the photodiode signal with and without sample in place, one can deduce the absolute power absorbed by the sample during irradiation. Using this system to irradiate a sample for a known time and measuring the absolute yield of chemical products (e.g. photometrically) gives rise to an absolute determination of the radiolytic yield under investigation (i.e. the one corresponding to the product measured). Details of the sample chamber are shown in Fig. 4. This is coupled to a sample delivery and flushing system, consisting of an airtight chamber with a cylindrical hole in a polyethylene terephthalate (PETE) block. Chamber o-rings and kapton windows are held together with PETE blocks on either side - the back PETE block connects the chamber to a support structure of the end station, with the chamber connected by polyether ether ketone (PEEK) tubing lines to and from the sample delivery and flushing system, the operation of which is described in the supplementary information.

### Irradiation and preparation

**Figure 5:**
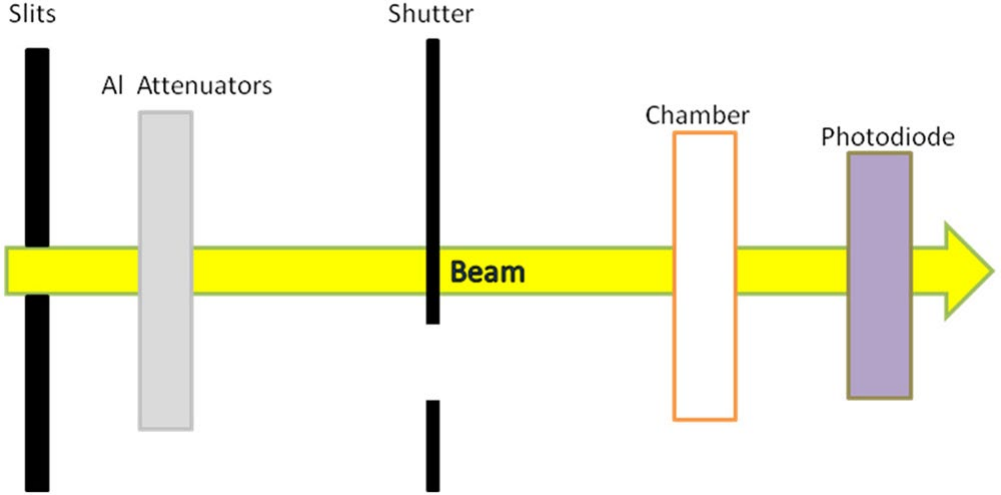
Schematic diagram of the set up on the beam line, after the monochromator and beam collimation system.

The ferrous sulphate dosimeter samples were irradiated at the B16 and I15 beamlines of the Diamond Light Source^29,31,32^. The samples (0.4 M sulphuric acid, 6 mM ammonium ferrous sulphate and 1 mM potassium chloride) were prepared with ultra-pure water and well-agitated to ensure that the solution is saturated with air, to insure that sufficient oxygen was present^5,14–16,18^. Figure 5 illustrates the irradiation geometry employed with a rectangular collimated X-ray beam. The apparatus was aligned so that the X-ray beam propagated through the sample chamber and other elements. Sheets of aluminium of varying thickness were used to attenuate the X-ray beam before it entered the sample chamber. These attenuators are used to reduce the intensity of the beam, and thus the dose rate. Behind the sample chamber a PD300-500CB photodiode (Canberra, United States)^38^, calibrated by the Physikalish-Technische Bundesanstalt, was set up to measure the power in the beam after propagating through the chamber. On B16, the photodiode was set 46.5 cm behind the chamber. On I15, the photodiode was set 162.5 cm behind the chamber. In both cases the absorption of X-rays in the air was taken into account as is described below. The shutter and an x-y translator were used to move different sample-catching wells under the ejection nozzle by a PC situated outside of the experimental hutch^19^. The shutter was controlled with electronic pulses that signaled when it should be open or closed. This means that the beam could only pass through the chamber at the above-mentioned times during the operation of the system. The length of time when the shutter is open with a sample in the chamber is recorded and associated with that sample. As noted above, there are times when the shutter is opened when there is no sample in the chamber. This enables the determination of the beam power with and without the sample present. The readings from the photodiode during these two set ups are used in the analysis to determine the energy deposited in the sample.

The Diamond Light Source’s EPICS-based data acquisition system^39^ was used to extract the readings from the photodiode and the concurrent current in the synchrotron ring (the ring current)^20^. These values were then recorded in a tab delimited text file, with the time at which they were taken. This text file is passed through a script to determine the power absorbed by the sample. Under irradiation the photodiode produces a current proportional to the power in the beam. The sensitivity of the photodiode is the ratio of the current produced to the power in the beam and it is calibrated for different energies of X-rays, to the Physikalish-Technische Bundesanstalt standards. This current is converted into a voltage through an amplifier; this voltage is the reading from the photodiode.

### Determination of Power Absorbed

The power absorbed by the sample is dependent on the power in the beam, during the irradiation. The power in the beam is proportional to the X-ray production of the synchrotron, which is dependent on the current in the synchrotron storage ring (electron beam ring current). Accordingly, a photodiode value equivalent to one produced by this power can be calculated from the readings without the sample, the baseline readings, and concurrent ring current. These baseline readings are divided by their concurrent ring current to calculate the average reading to ring current ratio, for the empty chamber. This ratio is used to get the equivalent reading that would be produced if the sample chamber were empty for any ring current. This is then used to get equivalent readings for the times when the sample is being irradiated, as in equation 6:

6 $${R}_{equiv}(t)=\langle \frac{{R}_{out}}{{I}_{ring}}\rangle \,\ast \,{I}_{ring}(t)$$

where *I*_*ring*_ (t) is the ring current measured at time *t* and *R*_*out*_ is the signal measured at the photodiode when there is no sample in the chamber (the baseline reading). The angle brackets represent the average taken over a long-time series (sufficiently long that the errors in this term are negligible) and *R*_*equiv*_(*t*) is the equivalent signal to *R*_*out*_(*t*) deduced from ring-current measurement, i.e. it can be determined even when sample is in the sample chamber. The power deposited in the sample is given by:

7 $${P}_{abs}=\frac{{e}^{-{\mu }_{en:air}x}-{e}^{-{\mu }_{en}x}}{{e}^{-{\mu }_{en}x}-{e}^{-\mu x}}\,\ast \,\frac{1}{{e}^{-{\mu }_{air}d}}\,\ast \,\frac{{R}_{equiv}(t)-{R}_{in}(t)}{S}$$

where *R*_*in*_ refers to the photodiode readings taken during irradiation while the sample is in the chamber, *S* is the product of the calibrated photodiode’s sensitivity and the gain of the amplifier used prior to digitization of the photodiode signal and *d* is distance from the chamber to the photodiode. Here *x* is the thickness of the sample chamber determined both by geometric measurement of the sample chamber and from the relationship:

8 $$x=\frac{-\mathrm{ln}(\langle \frac{{R}_{in}}{{R}_{equiv}}\rangle )}{\mu -{\mu }_{air}}$$

where *μ* and *μ*_*air*_ are the attenuation coefficients of the sample and air respectively, and *μ*_*en*_ and *μ*_*en*:*air*_ are the absorption coefficients of the sample and air respectively. These coefficients can be found in a database, maintained by NIST, developed from the ICRU reports^40^. The value of *x* determined from this is 4.06 ± 0.09 mm. All of the terms involving exponents represent small corrections for absorption of X-rays by the air. Errors in power absorbed and hence dose and dose rate were determined from a standard quadrature error propagation of equation 7. The dominant contribution to this error comes from the determination of *x* and the error in the calibrated diode’s sensitivity.

### Determination of G-Values

The ferrous sulphate dosimeter is based on quantifying the radiolytic oxidation of iron, the production of Fe^3+^ from Fe^2+^ by irradiation^5,14,15,18^. The amount of Fe^3+^ produced by irradiation is determined from its concentration in the irradiated sample. The concentration of Fe^3+^ was measured by taking an absorbance reading at 304 nm, and dividing by the difference between the molar attenuation coefficients of Fe^3+^ and Fe^2+^ at 304 nm^5,14,18^. Absorbance readings were taken with a 1800 spectrophotometer (Shimazdu, Japan).

The density of the ferrous sulphate dosimeters prepared for this paper, was measured as 1.025 ± 0.003 kg/L. The expected density for the ferrous sulphate dosimeter is 1.024 kg/L^5^. The sample masses were determined to be 146 ± 5 mg for the ferrous sulphate dosimeter described below, correspond to an average volume of 142 ± 5 *μ*l.

This volume of the sample was multiplied by the concentration of Fe^3+^ to calculate the amount produced during irradiation. This amount divided by the energy absorbed, during its production, gives the G-value for Fe^3+^. Note that although the irradiated volume is considerably less than the total sample volume, provided the production increases linearly with increased energy deposition, the G-value is reliably determined with the method described above. The linearity of production is discussed below. The axis of the chamber may be at a small angle to the beam. This would not affect the G-value measured, because this would only negligibly increase the relative pre-sample air attenuation of the beam on one side of the chamber. The mass of the sample, measured as described above, is then combined with the density of the sample to calculate the volume of the sample. The volume of the sample was multiplied by the concentration of the end product to calculate the amount of end product in the irradiated sample. Many such measurements were made for different irradiation times. An example of the resultant data is illustrated in Fig. 2.

A computer script was written to determine times when the shutter is open by looking for groups of photodiode readings with low variation (i.e. one opening and closing of the shutter gives rise to a top-hat function in the photodiode signal). These readings are separated into those taken while the sample is in the chamber and those taken when the chamber is empty using the magnitude of the readings. The power attenuated due to the sample from the beam is determined by taking the photodiode reading difference between these two states and converting it with the sensitivity of the photodiode to an absolute power. This value was then corrected for the attenuation of the beam by the air between the chamber and the photodiode, according to equation 7, and converted to total energy with a record of the irradiation time. This record can then be verified by comparison to the record of photodiode signal.

### Determination of the average dose rate during X-ray pulses in the irradiated volume

Although the G-value does not depend on the volume of sample irradiated, the instantaneous dose rate in the sample does. The power absorbed can also be used with the mass of the sample to calculate the average dose rate. The volume irradiated was calculated by multiplying the area of the beam by the thickness of the sample from equation 8. The area of the beam can be taken from the set size of the slits. It was also empirically determined by measuring the burn marks on a piece of EBT3 Gafchromic film^39^ irradiated at the front of the sample chamber. The area was found to be 6.4 ± 0.2 mm^2^, by both methods. The volume irradiated is then given by:

9 $${V}_{irrad}={A}_{beam}\,\ast \,x$$

where *V*_*irrad*_ is the volume that is irradiated by the beam, *A*_*beam*_ is the area that the beam projected perpendicular to the beam axis and the thickness of the sample is *x* (as defined above in Equation 8). The volume irradiated in the ferrous sulphate dosimeter was 25.6 ± 0.2 *μ*l on B16, and 24.8 ± 0.3 *μ*l on I15. The dose rate determined from the power absorbed is an average over many pulses. However, the synchrotron is not a continuous source of photons. X-rays are produced when bunches of electrons in the synchrotron ring are accelerated in a bending magnet or wiggler producing a train of X-ray pulses. Accordingly, the sample absorbs energy only during these pulses. The average time structure of the electron bunches was measured by a streak camera, confirming a Gaussian distribution centered at 93 ps with a FWHM of approximately 55 ps. On I15 a wiggler was used to generate the X-rays^20,32,41^. This means that the X-rays are produced by the electron bunches traveling through a series of magnets. Because the X-rays produced at the start of the wiggler travel faster than the electrons producing the X-rays, the time structure of the X-rays is slightly expanded in time. The distance between the X-rays produced at the start of the wiggler determines the pulse length extension time. Electrons move at 0.999997195c through the I15 wiggler which is 147 cm long^20,32,41^. Over the length of the wiggler the distance between the electron bunch and the first produced X-rays grows to 4 microns extending the pulse by 13 fs; this is a negligible increase in the pulse length. Accordingly, the X-ray pulses have the same time structure as the electron bunches. In a single revolution of electrons in the synchrotron there is a train of 900 bunches separated by 2 ns intervals and then a 72 ns pause and then the pattern repeats^20,32^. Each bunch has a full width at half maximum (FWHM) length of 43.36 ± 0.02 ps. The time structure of the bunch gives the time structure of the production of X-ray pulses. This means that during irradiation the sample absorbs energy during the pulse. The amount of energy absorption is proportional to the intensity of X-ray production, which is driven by the intensity of the electron bunch. The equivalent length of actual irradiation is 39.02 ± 0.09 ns in every 1.873 *μ*s of irradiation, which means the average dose rate during a pulse is 48.01 ± 0.11 times the average dose rate integrated over many pulses (i.e. as seen by the photodiode)^38^. The instantaneous dose rate varies within a pulse, however in order to meaningfully study the variation of G-value with dose rate, it is necessary to define an average dose rate in the pulse. We define this average power as the total energy delivered in a pulse divided by the FWHM time of the pulse.

## Conclusions

An accurate technique to determine absolute G-values for radiolytic processes with direct reference to the physical standards (through the use of a calibrated photodiode, weighing scales and volumetric flasks) is described. Although applicable to a wide range of photon or particle beam delivery systems, its use with synchrotron beams has been illustrated. This technique has been used to generate a new benchmark measurement, which could test radiation chemistry simulation codes^8–13^ over a wide range of dose rates. This technique produces an extremely accurate measure of the radiolytic yield of a product.

## Electronic supplementary material

Supplementary Information
