# Supplementary material for: Observation of dose-rate dependence in a Fricke dosimeter irradiated at low dose rates with monoenergetic X-rays
Source: Sci Rep. 2018 Mar 16;8:4735. doi: 10.1038/s41598-018-21813-z (PMC5856745; doi:10.1038/s41598-018-21813-z)
Supplement: Supplementary file 1 — Supplementary Information [file 41598_2018_21813_MOESM1_ESM.pdf]

Observation of dose-rate dependence in a Fricke dosimeter irradiated at low dose rates with monoenergetic X-rays, Supplementary Information

Mel O'Leary[1,2,*], Daria Boscolo[3], Nicole Breslin[1,2], Jeremy MC Brown[1,4], Igor Dolbnya[5], Chris Emerson[1,2], Catarina Figueira[1,2], Oliver JL Fox[5], David Robert Grimes[1,2,6], Vladimir Ivosev[1,7], Annette K. Kleppe[5], Aaron McCulloch[1,2], Ian Pape[5], Chris Polin[1], Nathan Wardlow[1], and Fred J Currell[1,2]

[1]School of Maths & Physics, Queen's University Belfast, University Road, Belfast, BT7 1NN, UK
[2]Centre for Advanced and Interdisciplinary Radiation Research (CAIRR), Queen's University of Belfast, BT7 1NN, Northern Ireland, UK
[3]GSI Helmholtzzentrum für Schwerionenforschung GmbH, 64291 Darmstadt, Germany
[4]Department of Radiation Science and Technology, Delft University of Technology, Delft, 2629 JB, The Netherlands
[5]Diamond Light Source Ltd., Harwell Science and Innovation Campus, OX11 0DE, Didcot, UK
[6]Cancer Research UK/MRC Oxford Institute for Radiation Oncology, Gray Laboratory, University of Oxford, Old Road Campus Research Building, Off Roosevelt Drive, Oxford OX37DQ, UK
[*]moleary05@qub.ac.uk

ABSTRACT

Operation of the measurement system and background data used to evaluate the role of possible artifacts are presented.

More details about the apparatus

Operation protocol

Sample Delivery and Flushing System

This automated delivery / flushing system (consisting of polytetrafluoroethylene tubing, syringes, stepper motors and valves) fills the chamber with sample prior to irradiation and then flushes the sample to be collected for analysis. Two small syringes (typically 3 ml for precision) attached to stepper motors drive sample through the system. The driver syringe contains sample, whilst the other contains air for flushing. These syringes are connected into the system by lines with in-pinch control values, connected by Luer-lok fittings to the syringes, as depicted in figure S1. Valves are set to either open or closed, controlling the flow of sample and air through the system. All valves are initially closed except valve 2. The driver syringe initially has its plunger removed to allow the air in the lines to be pushed out. Once the line is full, and the syringe is loaded with sample, a small meniscus is expressed from the top of the syringe and then the plunger is replaced and connected to a linear actuator, forming a syringe pump. In this manner no air is introduced. Table S1 sets out the different stages the system, a schematic representation is shown in figure S1. Once the lines and driver syringe are full, valves 1 and 3 are opened to push the sample through until the lines are full up to valve 3. With the lines full up to valve 3, both valves 2 and 3 are closed. Then air is pushed out through the chamber and valve 5 to clear the chamber of any aqueous solution that might be in it before use. At this point the system is in a ready state and able to begin an automated run.

The first step in the irradiation procedure is to fill the chamber with sample. In order to fill the sample chamber valves 1, 2 and 3 are opened; valve 5 is closed, as can be seen in the fill stage diagram of figure S1 and table 1. This setup allows sample to be pushed from the driver syringe into the chamber through valve 3. The open connection to atmosphere through valves 1 and 2 allows the air in the chamber to be displaced by sample out of the system. The sample moves into the chamber through the PEEK inlet nozzle. When the chamber has been filled, the driver syringe is pulled back until the sample in the line into the chamber is at its starting position. This leaves an air bubble at the top of the chamber, as is shown in the X-ray image of figure S2. The sample is left in the chamber during irradiation as depicted within the irradiation stage diagram of figure S1 and table S1. A shutter is used to control the irradiation time of the sample set via the user in the system control software. After the desired irradiation time is reached the shutter is closed and the sample is flushed out of the chamber through the ejection nozzle. To achieve this, the chamber is flushed with air with valve 1 and 5 open and valves 2 and 3 closed, as seen in the the flush stage

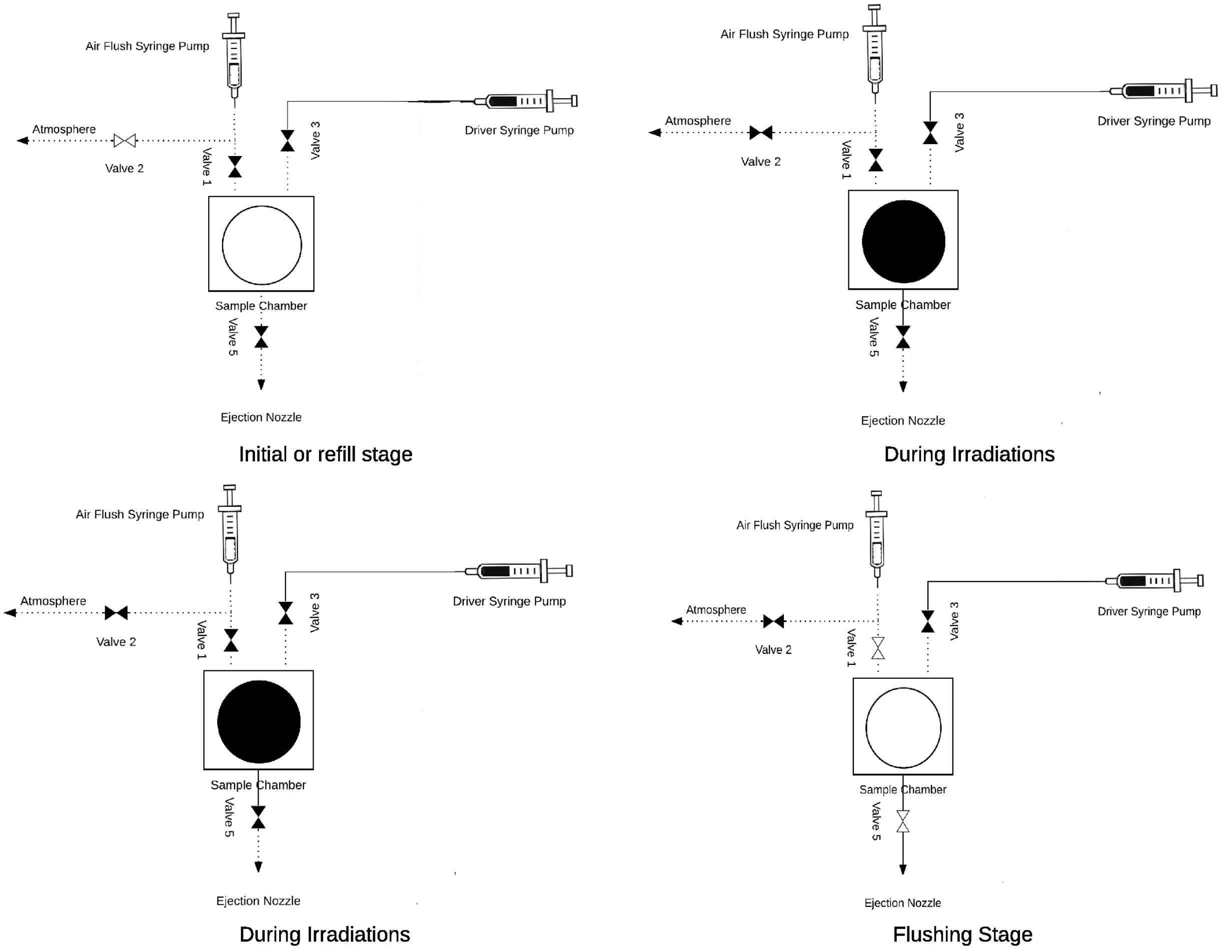

Figure 1. Diagrams of the sample chamber connected to the valve network of the sample delivery/flushing system. Solid lines indicate lines with sample in them and doted lines indicate lines with only air in them. Black valves are closed and white valves are open.

diagram of figure S1. The air flush syringe is pushed down, forcing air into the sample chamber, thereby flushing the irradiated sample through valve 5 and out the ejection nozzle, where it is collected for later analysis.

When the air syringe no longer contains enough air to fully flush a sample charge, a refill procedure is used, as depicted in figure 1 and table 1. For this procedure valve 1 and 5 are closed and valve 2 is opened. The air flush syringe is pulled back until it is full of air. Then the valves are returned to their original positions and the chamber is flushed with air again, this process is repeated again after the syringe has been emptied of air, until the chamber has been flushed three times. During the final refill, the shutter is opened again to get baseline measurements for the dose calculations described above. The system is now ready for another irradiation. This fill-irradiate-flush process is repeated to study the dose dependence on sample formation and to provide measurement replicates. A complete run of 20 irradiations takes about 3 ml of sample; typically quadruplicate measurements at the same dose are taken, giving 5 separate doses per syringe fill. Prior to irradiation, the mass of samples held within the chamber during irradiation is determined by pre-weighing a series of Eppendorf flasks, filling each with one charge from the system, and then reweighing them. The mass determined by this method is divided by the ferrous sulphate dosimeter's density, giving a determination of the sample's volume, given above.

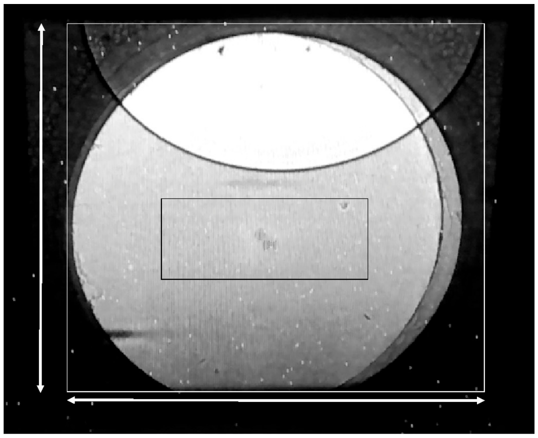

Figure 2. Screen capture from a video taken using an 'X-ray eye' of the sample chamber. When it's in the configuration that is used to irradiate samples. In order to capture this image, the X-ray beam was made much taller and wider than it would be for measurement. Lines with arrows indicate scale of 0.5 cm in the image. Outline box indicates the X-ray beam size for this image. The black box in the middle indicates the size of the beam during irradiations. The full circle in the image indicates the entrance/exit windows of the chamber, as can be seen there is a small tilt in the chamber. The arc of a circle coming in from the top of the figure shows the sample-air interface. The reproducibility of this sample configuration was confirmed by watching several fill/flush cycles with a large X-ray beam size (i.e. the configuration used to take this image).

STAGE	Valve 1	Valve 2	Valve 3	Valve 5	Shutter	Notes
Initial	CLOSED	OPEN	CLOSED	CLOSED	CLOSED	Default state for system
Filling	OPEN	OPEN	OPEN	CLOSED	CLOSED	Sample driven into chamber
Irradiation	CLOSED	CLOSED	CLOSED	CLOSED	OPEN	Sample is irradiated
Flushing	OPEN	CLOSED	CLOSED	OPEN	CLOSED	Sample flushed out and collected
Refill	CLOSED	OPEN	CLOSED	CLOSED	OPEN	Baseline readings taken

Table 1. Sequence of states that the camber goes through during a run.

Comparison between 6 mM Fe^{2+} and 10 mM Fe^{2+}

On I15 beam the radiolytic production of Fe^{3+} ions in a 6 mM formulation of the ferrous sulphate dosimeter (Fricke dosimeter) and a 10 mM formulation of the ferrous sulphate dosimeter were compared, and found to have similar dose response. The increase in concentration of Fe^{3+} for a given dose (energy absorbed per unit mass of sample), as can be seen in figure 3. This dose response of these samples gives a radiolytic yield of 1.19 ± 0.07 μmol/J for the 6 mM formulation, and 1.12 ± 0.13 μmol/J for the 10 mM formulation. The method to obtain these results was similar to the method used for the main results. The samples where place on the beam-line in cuvettes, with a thickness of 10 mm. The samples where irradiated for a different lengths time. The dose rate was calculated using the dosimetric methods outlined in this paper, multiplying these dose rates with the irradiated time, as the dose to each sample.

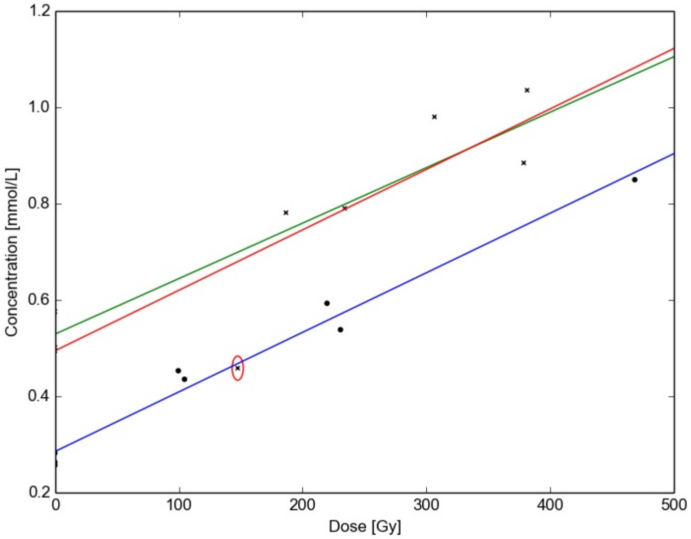

Figure 3. Plot of the concentration of Fe^{3+} against dose absorbed. The dots plot results from 6 mM ferrous ion concentration Fricke, and a linear fit to the 6 mM results is indicated by the blue line. The crosses plot results from 10 mM ferrous ion concentration Fricke, and the fit to the 10 mM results is indicated by the red line. The cross circled in red is an outlier result (it may not have been irradiated). The green line indicates the fit to the 10 mM results ignoring this point. For the 6mM results the radiolytic yield determined was 1.19 ± 0.07 μmol/J.For the 10mM results the radiolytic yield determined was 1.22 ± 0.23 μmol/J, and ignoring the outlier 1.12 ± 0.13 μmol/J.

Unattenuated X-ray spectrum

The unattenuated X-ray spectrum coming from the B16 beamline was calculated using XOP v2.4 (X-ray Oriented Programs) software package.[8] The power spectrum from a Diamond Light Source dipole bending magnet (electron energy 3 GeV, magnet field 1.4 T) was multiplied by the square of the calculated reflectivity of one of the multilayer monochromator mirrors, the square being used because there are two such mirrors in the beamline with beam being reflected off of each in turn. This spectrum was then used as the source term $S(E)$ in modeling effect of spectral hardening on the G-values measured. Note the intensity is shown on a logarithmic scale and the intensity of the harmonic (at 40 keV) is over two orders of magnitude lower than that of the main peak.

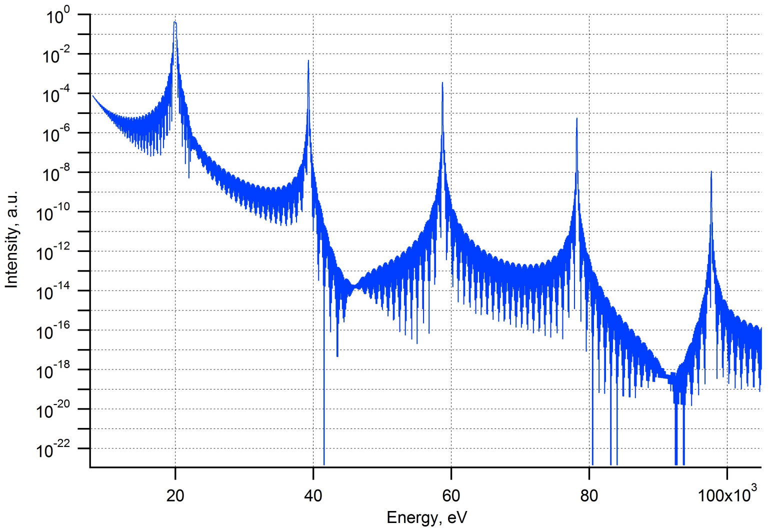

Figure 4. Calculated energy spectrum for Diamond Light Source beamline B16 when used with its multilayer monochromator tuned to pass an energy of 20 keV

Oxygen depletion model

The radiochemistry of Fricke dosimeters is deeply complex with over 60 known potential interactions[1,2]. Amongst these, there is a well-known oxygen dependent pathway, given by

$$e^-_{aq} + H^+ \rightarrow H^\bullet \tag{1}$$
$$H^\bullet + O_2 \rightarrow HO_2^\bullet \tag{2}$$

When no oxygen is present the number of Fe^{2+} ions radiolytically oxidized is reduced from three to one. This can act to markedly decrease the radiolytic yield, which after irradiation with Co-60 is typically 15.5 $\pm$ 0.2 ions per 100 eV versus only 8.2 $\pm$ 0.3 ions per 100 eV in an anoxic solution[2]. As each oxygen interaction depletes a single oxygen molecule, we should we then ask whether this depletion might explain the falling radiolytic yield observed in this work, and if not, eliminate it as a potential explanation the observed effect. We can investigate this using previous methods - firstly, given the proton radical diffusion constant in water and approximate mean-life time ($D = 7 \times 10^{-9}$ m^{2}s^{-1}, $\tau_e \approx 10^{-3}$s)[3], we can follow a previous similar approach[4] and estimate the thermal velocity of radicals, the mean-free path and the number of expected collisions, given respectively by

$$v_T = \sqrt{\frac{8k_BT}{\pi m_p}} = 2.4688 \times 10^3 m/s \tag{3}$$
$$l_f = \frac{3D}{v_T} = 8.445 \times 10^{-12} m \tag{4}$$
$$\eta_r = \frac{v_T^2 \tau_e}{3D} = 2.994 \times 10^{11} \tag{5}$$

where m_p is the radical mass, taken to be the same as a proton. As previously shown, the ratio μ_o of the number of oxygen molecules in a volume of water of oxygen partial pressure p_o over the number of water molecules in the same volume is given by

$$\mu_o = \left(\frac{\rho_w m_w}{\Omega m_{O2}}\right) p_o \tag{6}$$

where ρ_w is the density of water, Ω a known constant related to oxygen diffusion in water, m_w the mass of a water moleculate

and m_{O2} the mass of an oxygen molecule, all as previously estimated. Each Fricke oxygen pathway event depletes an oxygen molecule. Assuming no external re-oxygenation, we would expect this to gradually reduce the available oxygen in the dosimeter. The mean free potential of water can be taken as $I = 72\text{eV}$[5], and to a first approximation assuming all dose is absorbed, Ionization density I_D (defined as maximum ionizations per unit volume per unit time) is related to dose rate D_R by

$$I_D = \frac{D_R \rho_W}{I}. \tag{7}$$

Thus for a constant dose rate given for time t, we expect the ratio of oxygen molecules to water molecules in any given volume to be given by

$$\mu(t) = \mu_o - \frac{\sigma_o I_D m_m t}{\rho_w}. \tag{8}$$

where m_m is the mass of a single water molecule, and σ_o the probability of the oxygen-dependent interaction occurring. Taking this ratio as a probability, we can then state the total probability of an interaction between available oxygen and this hydrogen species, simply by writing

$$P(t) = 1 - (1 - \mu(t))^{\eta_r} \tag{9}$$

and the minimum time to oxygen depletion to be

$$t_D = \frac{\rho_w \mu_o}{\sigma_o I_D m_m}. \tag{10}$$

However, it is important to note that oxygen depletion does not explain the observed dose rate effect seen in the current work; as illustrated in figure 5, whilst oxygen depletion is linear, simulations suggest a rapid switch from to non-oxic pathways when oxygen is depleted rather than a gradual decline, in agreement with literature to date[6,7]. Crucially, oxygen depletion is dose dependent rather than dose rate dependent, and can be excluded as the cause of the dose rate effect observed in this work.

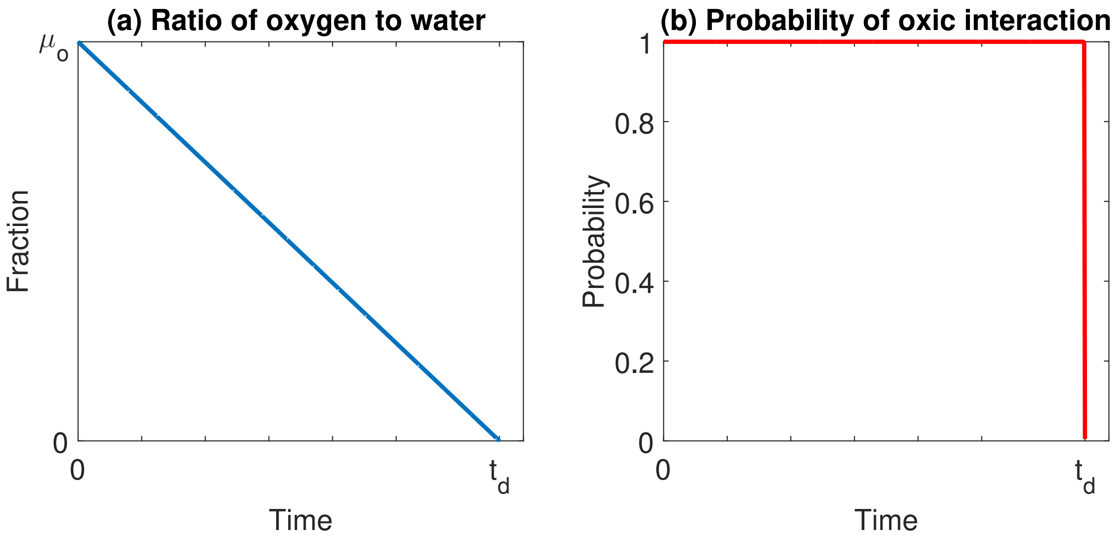

Figure 5. (a) Oxygen depletion with time is linear (b) The probability of a radical encountering an oxygen molecule remains almost unity until oxygen is virtually extinguished. Importantly, there is no apparent dose rate dependence on oxygen depletion, only total dose dependence.

References

1. Meesat, R. *et al.* Utilization of the ferrous sulfate (fricke) dosimeter for evaluating the radioprotective potential of cystamine: experiment and monte carlo simulation. *Radiat. research* **177**, 813–826 (2012).
2. McAuley, K. & Nasr, A. Fundamentals of gel dosimeters. In *Journal of Physics: Conference Series*, vol. 444, 012001 (IOP Publishing, 2013).
3. BioNumbers Diffusion coefficient of proton. In *Harvard BioNumbers database*, http://bionumbers.hms.harvard.edu//bionumber.aspx?id=106702&ver=13 (Harvard, 2011).
4. Grimes, D. R. & Partridge, M. A mechanistic investigation of the oxygen fixation hypothesis and oxygen enhancement ratio. *Biomed. physics & engineering express* **1**, 045209 (2015).
5. Grimes, D. R. & Warren, D. R. Partridge, M. An approximate analytical solution of the Bethe equation for charged particles in the radiotherapeutic energy range. *Nat. Sci. Reports* **7**, 9781 (2017).
6. Spinks, J. W. T. & Woods, R. J. *An introduction to radiation chemistry* (1990).
7. Klassen, N., Shortt, K., Seuntjens, J. & Ross, C. Fricke dosimetry: the difference between g (fe3+) for 60co-rays and high-energy x-rays. *Phys. medicine biology* **44**, 1609 (1999).
8. ESRF X-ray Oriented Programs software package. http://www.esrf.eu/Instrumentation/software/data-analysis/xop2.4. Accessed: 2017-12-08.
